# Hammerhead Ribozymes: Structural Insights, Catalytic Mechanisms, and Cutting-Edge Applications in Synthetic Biology

**DOI:** 10.3390/ijms26125624

**Published:** 2025-06-12

**Authors:** Liangliang Wang, Yan Liu, Xuemin Xian, Haitao Zhang

**Affiliations:** 1 Department of Biochemistry and Molecular Biology, Guangdong Medical University, Zhanjiang 524023, China; liuy598@mail2.sysu.edu.cn (Y.L.);; 2 School of Biological and Pharmaceutical Engineering, Lanzhou Jiaotong University, Lanzhou 730070, China; 3 MOE Key Laboratory of Bioinorganic and Synthetic Chemistry, School of Chemistry, Sun Yat-sen University, Guangzhou 510275, China

**Keywords:** hammerhead ribozymes, RNA catalysis, gene regulation, antiviral therapy, biosensing

## Abstract

Hammerhead ribozymes are a class of small RNA molecules with catalytic activity. Their compact size, high catalytic efficiency, structural simplicity, and modular design flexibility make them ideal tools for RNA manipulation and gene regulation. In recent years, these ribozymes have demonstrated tremendous application potential across diverse fields, including gene regulation, disease therapy, and biosensing, significantly advancing related research. This article provides a comprehensive review of recent progress in hammerhead ribozyme research within synthetic biology, thoroughly examines the current challenges, and outlines future development directions, aiming to offer valuable perspectives and insights for their biomedical applications.

## 1. Introduction

Ribozymes are a class of RNA molecules with catalytic activity that can catalyze specific chemical reactions [1,2]. Their discovery challenged the long-held belief that “all enzymes are proteins” and highlighted RNA’s pivotal role in life’s origin and evolution [3,4,5,6]. Since Cech and Altman independently discovered ribozymes in 1982 and were awarded the Nobel Prize in Chemistry in 1989, ribozyme research has become a fundamental area of molecular biology [7,8,9,10,11,12,13,14].

**Figure 1:**
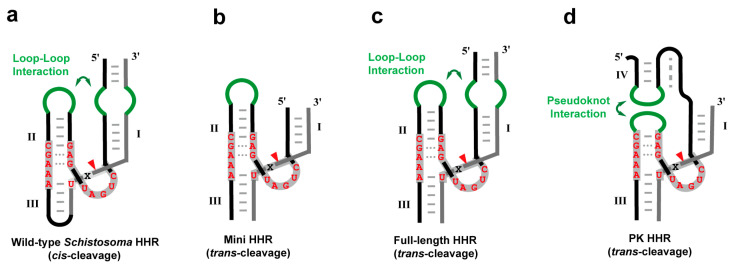
Secondary structure schematics and catalytic core sequences of hammerhead ribozyme (HHR) variants. (**a**) Wild-type *Schistosoma* HHR; (**b**) minimal HHR (Mini-HHR); (**c**) full-length HHR; (**d**) pseudoknot-containing HHR (PK-HHR). The conserved catalytic core sequence is highlighted in red. The cleavage site is indicated by a red arrow, with “X” denoting A, C, or U nucleotides.

Among the known ribozymes, the hammerhead ribozyme was one of the most thoroughly studied types due to its small size, simple structure, and high degree of modularity [15,16,17,18]. It was initially discovered in plant viroids and satellite RNAs [19,20], and subsequent research has shown that it is widely present in almost all life systems, from prokaryotes to eukaryotes [16,21,22,23,24]. However, natural hammerhead ribozymes primarily exhibit self-cleavage (*cis*-cleavage) activity, and their efficient intracellular catalytic function relies on the tertiary interactions formed between the stem II-loop and the bulge structure of stem I (Figure 1a). While this ensures efficient self-cleavage, it restricts their utility as *trans*-acting catalysts.

The structural engineering of hammerhead ribozymes has yielded functional variants with *trans*-cleavage activity (Figure 1b) [25]. By modifying stem I and stem III sequences while preserving the conserved catalytic core, these variants can specifically cleave RNA at NUH sites (where N is any nucleotide, and H is A, C, or U) [26,27]. This approach endows hammerhead ribozymes with great flexibility, theoretically allowing them to target any RNA-containing NUH sites. Such innovations have expanded their applications and provided novel tools for gene function studies and therapy [28].

This review systematically summarizes the structural characteristics, the catalytic mechanisms, and the most recent breakthroughs in hammerhead ribozyme research, with a focus on their applications in gene regulation, antiviral therapy, and biosensing. Unlike previous reviews, this work highlights cutting-edge advancements, such as engineered *trans*-cleaving ribozymes, allosteric regulation strategies, and CRISPR-integrated systems, which have significantly expanded the toolset for synthetic biology and precision medicine. By critically analyzing current challenges and future directions, this article aims to not only provide a comprehensive reference for researchers in the field but also to provide insights into novel methodologies and therapeutic potentials, thereby fostering further innovation in the biomedical applications of hammerhead ribozymes.

## 2. The Structure and Catalytic Mechanism of the Hammerhead Ribozyme

### 2.1. Structural Characteristics of the Hammerhead Ribozyme

The hammerhead ribozyme is among the smallest known RNA-cleaving ribozymes. The naturally occurring wild-type *cis*-acting hammerhead ribozyme (wild-type HHR) comprises approximately 63 nucleotides (Figure 1a). Its minimal functional version (mini HHR) can be obtained by truncating stem regions and separating the ribozyme into independent enzyme and substrate strands, while maintaining nearly complete catalytic activity. The mini HHR consists of three helical domains (stems I, II, and III) surrounding a highly conserved core region of about 15 nucleotides, forming the characteristic “hammerhead” secondary structure (Figure 1b) [18,27,29].

Early structural studies primarily utilized this minimal version to investigate the hammerhead ribozyme’s structure and catalytic mechanism. However, research in the early 21st century demonstrated that tertiary interactions in full-length HHR can enhance reaction rates by more than 1000-fold compared to the minimal construct [30,31,32,33]. A landmark study in 2006 by Martick and Scott determined the crystal structure of the full-length HHR from *Schistosoma* mansoni, revealing key distant interactions: its catalytic core structure differs significantly from that of the minimal HHR, with stable tertiary interactions confirmed between the stem II-loop and the bulge structure of stem I (Figure 1c) [34]. Further work by Nelson and Uhlenbeck showed that the minimal HHR spends considerable time sampling inactive conformations, which explains its significantly reduced activity [33]. These findings shifted research focus toward hammerhead ribozymes retaining these distal tertiary interactions, which better reflect biological activity [35].

Recently, Breaker and colleagues identified a novel hammerhead ribozyme variant (PK HHR) through bioinformatics analysis. In this ribozyme, the loop corresponding to helix II forms a pseudoknot (PK) with an additional stem-loop connected to helix I (Figure 1d) [36]. This pseudoknot plays a similar role in the overall structure to that of helix II in the standard hammerhead ribozyme structure, and exhibits increased activity under standard conditions compared to other hammerhead ribozymes [36,37]. Interestingly, this pseudoknot-containing hammerhead ribozyme shares structural similarities with pistol ribozyme in its crystal structure, having an essentially identical secondary structure and connectivity, but its catalytic mechanism aligns with full-length HHR rather than pistol ribozyme [37]. Notably, despite the conserved core sequence among different hammerhead ribozyme variants, their helical regions show considerable variations in length and sequence depending on their biological sources [24], providing a convenient modularity for engineering applications.

### 2.2. Catalytic Mechanism of the Hammerhead Ribozyme

The RNA cleavage mechanism catalyzed by hammerhead ribozyme has been extensively studied [18,34,38,39,40,41,42,43], revealing a complex, well-ordered multistep reaction process comprising three main stages: substrate recognition, catalytic reaction, and product release. During substrate recognition, the hammerhead ribozyme specifically identifies and binds target RNA through the Watson–Crick base pairing in its stem regions, ensuring the precise targeting of RNA sequences. Upon substrate binding, the catalytic reaction initiates via general acid-base catalysis. In the catalytic core, the N1 position of G12 acts as a general base, deprotonating the adjacent ribose’s 2′-hydroxyl group (the nucleophile), while the 2′-OH of G8 functions as a general acid, donating a proton to the 5′-oxygen (O5′) at the scissile phosphate. This concerted acid-base catalysis drives the cleavage of the phosphodiester bond, generating 2′,3′-cyclic phosphate and 5′-hydroxyl terminal products.

Subsequently, the products are released from the active center of the ribozyme, allowing the ribozyme to enter another catalytic cycle. Throughout this process, metal ions—particularly Mg^2+^—play crucial roles [41,44,45,46,47,48,49]. High concentrations of Mg^2+^ can support the efficient cleavage activity of the minimal HHR (but not optimal cleavage) [50]. Moreover, other cations can also mediate cleavage reactions. For instance, the A6C variant of hammerhead ribozyme from the bacteriophage Bcep176, containing a single-point mutation in its core region, exhibits altered ion specificity with preferential selectivity for Mn^2+^ [51]. Additionally, organic cations like tetrapentylammonium ions [52,53] and spermidine [54] can significantly enhance catalytic activity as well.

## 3. Applications of Hammerhead Ribozymes in Synthetic Biology

### 3.1. Gene Regulation Applications

#### 3.1.1. RNA Switches Based on Hammerhead Ribozymes

**Figure 2:**
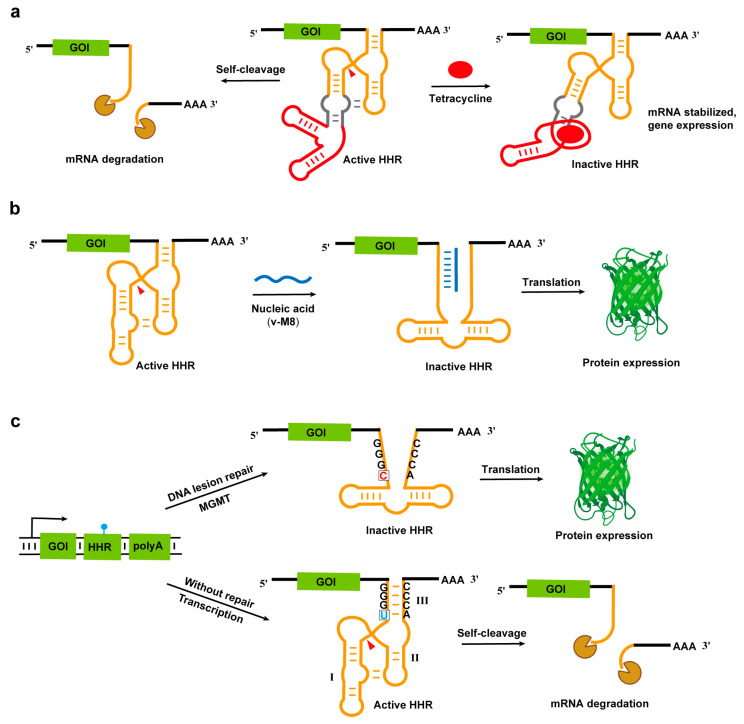
Hammerhead ribozyme-based RNA switches. (**a**) Schematic illustration of the tetracycline-inducible ribozyme ON-switch. A communication module (gray) links the hammerhead ribozyme to a tetracycline-binding aptamer (red). In the absence of the ligand, self-cleavage (red arrow) occurs, resulting in mRNA degradation. Tetracycline binding induces a conformational change that inactivates the ribozyme, leading to mRNA stabilization and subsequent gene expression. (**b**) Morpholino oligomer (PMO)-controlled hammerhead ribozyme switch. In this system, the hammerhead ribozyme functions as an OFF-switch for transgene expression, effectively suppressing target gene expression. Upon administration of a complementary morpholino oligomer (v-M8) into transgenic tissues, v-M8 specifically binds to and inhibits ribozyme activity, thereby restoring transgene expression (ON-switch). (**c**) DNA repair-mediated gene expression regulation strategy. A specific O^6^-MeG modification (indicated by the blue circle) was introduced into the transcribed strand of synthetic plasmids. Without repair, the plasmid produces active HHR with a fully matched stem III, leading to rapid mRNA degradation due to O^6^-MeG-induced transcriptional mutagenesis. MGMT-mediated DNA repair converts the misincorporated U to C in mRNA transcripts, creating a C:A mismatch in stem III. This mismatch inactivates HHR, stabilizing the mRNA and enabling protein expression. The cleavage site is marked by the red arrow, while GOI denotes the gene of interest.

The compact and modular nature of hammerhead ribozymes make them an ideal platform for constructing synthetic biology components, enabling researchers to develop diverse gene regulation systems. In recent years, a series of self-cleaving RNA switches based on hammerhead ribozymes have been developed. By incorporating them into the 3′ or 5′ untranslated regions (UTRs) of mRNA, the precise regulation of gene expression can be achieved. When a target gene’s 3′ terminus carries the hammerhead ribozyme sequence, the transcribed RNA spontaneously folds into a catalytically active conformation and undergoes self-cleavage. This process leads to polyA tail loss, resulting in transcript destabilization and rapid degradation, ultimately suppressing target gene expression (Figure 2). Notably, the self-cleavage activity of hammerhead ribozymes can be regulated by diverse inducers, including small molecules [55,56,57,58,59,60,61], nucleic acid [62], or proteins [63], among others. When these regulatory factors are present, they inhibit the ribozyme’s cleavage activity, thereby maintaining intact transcripts and preserving normal gene expression.

In Caenorhabditis elegans, Wurmthaler et al. successfully achieved precise gene expression regulation through a tetracycline-dependent hammerhead ribozyme-based RNA genetic switch system (Figure 2a) [64]. By inserting the ribozyme into the 3′ untranslated region (UTR) of target genes, any gene of interest could be converted into a tetracycline-inducible gene, thereby enabling the temporal control of gene expression across all developmental stages of the nematode.

As theophylline is an important small-molecule ligand, researchers have developed multiple theophylline-dependent hammerhead ribozyme aptamer variants through rational design and intracellular screening [60,65,66,67,68]. These variants have demonstrated regulatory capabilities in various fields, such as regulating the proliferation of mammalian T cells [69], the in vivo screening of caffeine demethylase activity [70], regulating the activity of the nuclease Cas9 to reduce off-target effects [71], and controlling gene expression and safety in the field of gene therapy based on adenoviruses [61,72,73]. Currently reported aptamer enzymes that function in prokaryotic or eukaryotic cells are mostly constructed by connecting different aptamers to the stem III region of the hammerhead ribozyme and achieving functional regulation in a *cis*-cleavage form [74]. Recently, Zhou et al. reported an intracellular screening method for hammerhead aptamer enzymes based on the toxin protein IbsC, successfully obtaining variants containing theophylline aptamers in the stem II region. Experiments have shown that theophylline can efficiently induce the corresponding *trans*-aptamer enzymes, effectively knocking down different target genes in eukaryotic cells [75]. The optimal T195 variant exhibits significant ligand dependence and dose dependence, and the cleavage efficiency can be further improved by introducing multiple aptamer enzymes.

Morpholino oligomers (PMOs) are DNA analogs where the pentose sugar ring is replaced by morpholine rings and phosphate groups are modified, enabling specific nucleic acid binding while resisting nuclease degradation. Researchers combined the transcriptional repression function of ribozymes with PMO-mediated ribozyme inhibition to create an efficient RNA switch system [62]. Here, the hammerhead ribozyme serves as an “OFF switch” suppressing transgene expression until complementary v-M8 morpholino oligomers inhibit ribozyme activity to “reactivate” expression (Figure 2b). This switch system enables the v-M8 dose-dependent regulation of protein expression, with the highest protein expression level being increased by up to 223 times, and this regulatory effect can be stably maintained for at least 43 weeks, demonstrating a wide range of gene expression regulation and long-term stability. Notably, the system has a relatively short induction half-life, but by replacing the octaguanidine dendrimer in the v-M8 morpholino oligonucleotide with a cell-penetrating peptide (CPP), the induction half-life can be significantly improved [76].

The integration of hammerhead ribozymes with gene-editing technologies has opened up new avenues for precise gene regulation. Jiang et al. designed and developed a ribozyme regulation system based on the DNA repair mechanism by inserting type III HHR sequences containing 8-oxoguanine (8-oxoG) or O^6^-methylguanine (O^6^-MeG) modifications into the 3′UTR of the target gene (GOI) (Figure 2c) [63]. Normally, the modified bases cause a stem III pairing that activates ribozyme cleavage, thereby blocking protein expression; however, in cells overexpressing MutY DNA glycosylase (MUTYH) or O^6^-methylguanine-DNA-methyltransferase (MGMT), DNA repair induces a mismatch in the stem III region, inactivating the ribozyme and restoring target gene expression. This cell-specific regulation strategy has been successfully applied to the CRISPR/Cas9 system, achieving the specific editing of the oncogene Plk1 and tumor growth inhibition in mouse models. Furthermore, Zhan’s CasRx-crRNA-ribozyme composite system (CCRS) integrated hammerhead ribozymes with Cas13 technology, outperforming conventional methods using CasRx, shRNA, or ribozyme alone in terms of mRNA/ncRNA knockdown efficiency [77].

In the field of synthetic biology, hammerhead ribozymes are not only used for the silencing of single genes but also show potential in the assembly of gene circuits. Researchers have developed protein-responsive ON-type switches and successfully constructed multi-step gene regulation cascades. Through self-cleaving hammerhead ribozymes, these switches can achieve an up to 20-fold upregulation in expression in response to specific proteins. This innovative strategy not only enables complex gene regulation but also provides new possibilities for constructing artificial cells with functions beyond those of natural cells [78].

#### 3.1.2. Engineering and Screening of Hammerhead Ribozyme-Based Gene Regulation Tools

Although hammerhead ribozyme-based switches represent promising therapeutic tools for manipulating target gene expression, the vast majority of hammerhead ribozymes exist in a self-cleaving form, making it difficult to achieve *trans*-cleavage of substrate RNAs. Moreover, their optimal intracellular activity depends on tertiary interactions between the stem II-loop and the bulge of stem I, which often limits their effectiveness in gene-silencing applications.

**Figure 3:**
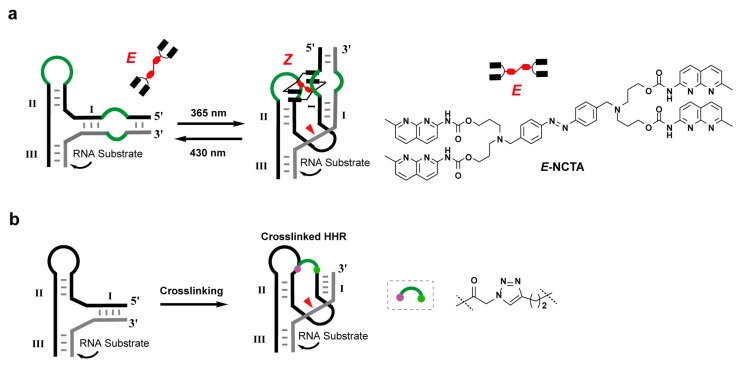
(**a**) Schematic illustration of photoactivatable hammerhead ribozyme (HHR) regulation via NCTA photoisomerization. The NCTA undergoes reversible *E*/*Z* photoisomerization upon light irradiation at 365 nm or 430 nm, enabling the optical control of ribozyme activity. (**b**) Schematic of the chemically cross-linked hammerhead ribozyme (crosslinked HHR). The red arrow indicates the targeted cleavage site within the substrate RNA.

In 2017, Mao’s research explored how small-molecule recognition triggers the tertiary interaction in hammerhead ribozymes. By using tris(2-aminoethyl)amine (tren)-derived scaffolds bearing two or four melamine rings to target oligo T/U domains in DNA/RNA, they successfully achieved ribozyme reactivation and allosteric regulation [79]. Subsequently, in 2019, Liang et al. demonstrated the role of RNA secondary and tertiary structural reorganization in catalysis, using synthetic bPNAs (bifacial peptide nucleic acids) as allosteric triggers to control HHR catalytic activity [80]. More recently, researchers developed a light-controlled hammerhead ribozyme system by taking advantage of the reversible regulation of secondary and tertiary RNA structures by the light-switchable molecular glue (NCTA) (Figure 3a) [81]. In NCTA-treated cells, the RNA cleavage activity of hammerhead ribozymes (which is dependent on proper RNA folding) can be reversibly regulated by light irradiation, achieving the precise optogenetic control of RNA structure and function.

Based on the critical regulatory role of distant interactions in the catalytic activity in the tertiary structure of hammerhead ribozymes, Wang et al. innovatively introduced intramolecular linkages into the minimal hammerhead ribozyme scaffold, successfully reconstructing the key distant interactions in the natural tertiary structure and developing a *trans*-acting hammerhead ribozyme variant with a chemically covalently cross-linked structure (Figure 3b). This modified ribozyme not only exhibits significantly enhanced RNA substrate cleavage efficiency but also overcomes the sequence limitations of traditional hammerhead ribozymes [82]. It has been demonstrated that this can efficiently regulate the expression levels of various exogenous and endogenous RNAs, showing broad application prospects as a novel gene therapy tool.

In addition to chemical modification approaches, Huang et al. established an intracellular screening system for *trans*-cleaving hammerhead ribozymes that innovatively combines a toxin-based reporter for directed evolution with a dual-fluorescent quantification assay to precisely evaluate intracellular cleavage activity. The optimized ribozyme variants obtained through this screening platform have demonstrated stable and significant gene suppression across multiple cell models [83].

### 3.2. Applications of Hammerhead Ribozymes in Antiviral Therapy

Hammerhead ribozymes, due to their ability to specifically recognize and cleave RNA target sequences, have shown broad application prospects in antiviral therapy. By designing hammerhead ribozymes targeting key regions of viral genomes, the replication and transmission of viruses can be effectively inhibited. In recent years, significant progress has been made in the research on the use of hammerhead ribozymes against various viruses, providing important ideas for the development of new antiviral therapies.

In the research on the novel coronavirus (SARS-CoV-2), researchers used bioinformatics tools such as RNABOB to successfully screen out 39 hammerhead ribozyme variants with high catalytic activity. In vitro characterization confirmed that these ribozymes could stably perform RNA cleavage functions under different metal ion conditions and precisely target the conserved regions of the SARS-CoV-2 genome, offering potential candidates for broad-spectrum coronavirus therapeutics [84]. Similarly, optimized hammerhead ribozymes against Chikungunya virus (ChikV) demonstrated potent inhibition in cellular models and significantly reduced viral loads in animal studies by blocking viral RNA replication [85]. Currently, hammerhead ribozyme technology has achieved good results in the prevention and control of various important human pathogens, including hepatitis B virus (HBV) [86], hepatitis C virus (HCV) [87], human immunodeficiency virus (HIV) [88], and dengue virus (DENV) [89], highlighting its versatile therapeutic potential.

Beyond direct antiviral therapeutics, hammerhead ribozymes also play an important role in fundamental virology research and vaccine development. A prime example is their application against Senecavirus A (SVA), an emerging pathogen that has significantly impacted global swine production in recent years. Researchers made a groundbreaking advancement by strategically inserting specific hammerhead ribozyme sequences at both termini of the viral genome, successfully establishing a reverse genetics-based virus rescue system. This innovative approach not only facilitates mechanistic studies of SVA replication and pathogenesis but also provides a platform for the development of novel antiviral agents and genetically engineered vaccines [90].

### 3.3. Applications of Hammerhead Ribozymes in Biosensing

Based on the programmability and sequence-specific recognition ability of hammerhead ribozymes, they can be combined with specific fluorescent markers or other reporting systems to develop biosensing tools for detecting various nucleic acid targets or biomolecules [91]. When hammerhead ribozymes bind to target RNA and undergo cleavage reactions, this leads to changes in fluorescence signals or other detectable signals, enabling the rapid and sensitive detection of specific molecules, which can be applied in disease diagnosis, environmental monitoring, and other fields.

**Figure 4:**
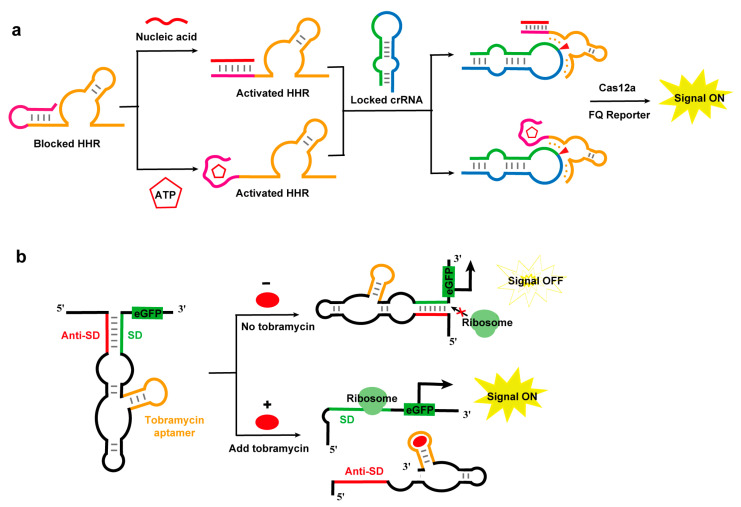
Hammerhead ribozyme-based biosensors: (**a**) allosteric ribozyme-driven crRNA switch biosensor, showing a schematic illustration of a biosensor design for the ultrasensitive detection of nucleic acid (miR-155) and ATP. The red arrow indicates the cleavage site; (**b**) tobramycin-dependent sensor strategy. In the absence of the ligand, the formation of the aptamer domain induces structural rearrangement, thereby suppressing the catalytic conformation of the ribozyme. Conversely, ligand binding to the aptamer triggers liberation of the Shine–Dalgarno (SD) sequence, activating gene expression and producing a “signal-on” fluorescent response in cells.

Recently, researchers have developed a CRISPR/Cas12a-based sensor employing hammerhead ribozyme-driven crRNA switches for highly sensitive biomolecule detection (Figure 4a). This innovative system utilizes the allosteric regulation properties of hammerhead ribozymes: target molecules specifically induce conformational changes in blocked ribozyme modules, restoring their RNA cleavage activity. The activated ribozyme then precisely recognizes and cleaves self-blocked inactive crRNA, releasing fully functional crRNA to activate the CRISPR/Cas12a system for the amplification and reporting of signals. Based on this principle, this sensor successfully achieved the ultrasensitive detection of miR-155 and ATP, with limits of 256 fM and 160 nM, respectively [92]. Notably, this sensor can accurately quantify the expression level of miR-155 in cancer patient cells and serum samples, fully demonstrating its application potential in clinical diagnosis.

Furthermore, hammerhead ribozymes have been employed to construct whole-cell microbial sensors that are responsive to specific molecules. For instance, researchers developed a highly sensitive microbial sensor for broad-spectrum antibiotics (e.g., tobramycin) by coupling an antibiotic-binding aptamer with a hammerhead ribozyme [93]. The design principle of this sensor is to incorporate the self-cleaving hammerhead ribozyme (HHR) N79 from *Schistosoma* mansoni into the 5′ untranslated region (5′UTR) of mRNA. In the absence of ligands, aptamer domain formation induces structural rearrangements that inhibit the ribozyme’s active conformation. Upon ligand–aptamer binding, the ribosome binding site (RBS) sequence is released, creating a “signal-ON” effect in cells (Figure 4b). This sensor maintains excellent stability in complex environments while enabling rapid antibiotic detection with a remarkable detection limit of 30 nM, well below the EU’s maximum residue limit [93].

The application of hammerhead ribozymes in biosensors demonstrates their tremendous potential in precision detection, their rapid response, and their multifunctionality. With further research, hammerhead ribozymes are expected to play increasingly important roles in clinical diagnostics, environmental monitoring, food safety, and other fields. For example, in food safety testing, hammerhead ribozyme biosensors can be designed to target specific pathogenic microorganisms or harmful chemicals, enabling the rapid on-site detection of contaminants in food and effectively ensuring food safety. In environmental monitoring, beyond antibiotic residue detection, these tools could be extended to identify other contaminants, such as heavy metal ions and organic pollutants, providing more comprehensive and efficient technical solutions for ecological environment assessments.

## 4. Conclusions and Perspectives

Hammerhead ribozymes, as catalytic RNA molecules, have become indispensable tools in synthetic biology and gene regulation due to their structural simplicity, high catalytic efficiency, and modular design. This review summarizes their structural characteristics, catalytic mechanisms, and diverse applications in gene regulation, antiviral therapy, and biosensing. In the field of gene regulation, engineered hammerhead ribozymes not only can achieve *trans*-cleavage activity but also respond to various inducers including small molecules, nucleic acids, and proteins, opening new avenues for precise gene expression control. In antiviral applications, studies using them to target SARS-CoV-2, HIV and other viruses demonstrated their therapeutic potential. In biosensing, innovative designs have enabled the highly sensitive detection of nucleic acids and biomolecules. Additionally, recent findings show that hammerhead ribozyme activity and stability can be modulated by external factors like lipid membranes [94,95], suggesting the possibility of novel membrane-based RNA biosensors and further expanding their application scope. These advances have significantly enhanced our understanding of hammerhead ribozyme functions and established a solid foundation for their wide application in the biomedical field.

Despite the significant progress in hammerhead ribozyme research, many challenges remain. Through a systematic comparison of natural ribozyme variants, researchers have found that certain mutations in the catalytic core do not always destroy their activity [21], challenging conventional wisdom and suggesting that our understanding of ribozyme evolution and function still needs to be deepened. Secondly, the stability and specificity of hammerhead ribozymes in cellular environments still need to be optimized. For example, interference from non-target RNAs may lead to off-target effects, while degradation by intracellular nucleases can reduce their activity. Moreover, the lack of efficient delivery systems also hinders their practical application in gene therapy. To address these challenges, researchers have actively explored multiple strategies: (1) in vivo directed evolution to select variants better adapted to cellular environments; (2) chemical modifications like D-ribose substitutions [96] to enhance nuclease resistance; and (3) structural engineering including chemical crosslinking [82] to maintain active conformations and improve catalysis. Importantly, the introduction of artificial intelligence and machine learning technologies has created new opportunities in hammerhead ribozyme research [97,98]. With these cutting-edge technologies, it is possible to more efficiently analyze massive ribozyme sequence and structure data, predict ribozyme functions, and design more intelligent ribozyme variants to meet the diverse needs of complex biological systems.

Looking forward, hammerhead ribozyme research will increasingly focus on interdisciplinary integration and practical applications. Fundamental studies should elucidate their catalytic mechanisms and structural dynamics, particularly how diverse core mutations affect function, to inform rational design. Applied research could combine hammerhead ribozymes with gene editing technologies like CRISPR/Cas to achieve breakthroughs in cancer treatment and genetic disorder correction [77]. Expansions of their applications in environmental monitoring and food safety as these fields gain importance also show promise. Through continued innovation and collaboration, hammerhead ribozymes are poised to play pivotal roles in precision medicine and synthetic biology, offering new opportunities to advance human health and biotechnology.

## Data Availability

No new data were created or analyzed in this study.
